# Multimodal Physical Therapy Management of Dysmenorrhea With Coexisting Mechanical Low Back Pain and Early Degenerative Lumbar Spondylosis: A Case Report

**DOI:** 10.1002/ccr3.73215

**Published:** 2026-07-23

**Authors:** Nadia Afrin Urme, Asma Islam, Fabiha Alam, Md Rifat Haidar, Md Waliul Islam, Ranu Islam, Polok Halder

**Affiliations:** ^1^ Department of Physiotherapy Bangladesh Health Professions Institute, CRP Dhaka Bangladesh; ^2^ International Centre for Diarrheal Disease Research, Bangladesh (Icddr,b) Dhaka‐ Bangladesh; ^3^ Dhaka College of Physiotherapy Dhaka Bangladesh; ^4^ Department of Physiotherapy and Rehabilitation Jashore University of Science & Technology Jashore Bangladesh

**Keywords:** case report, dysmenorrhea, early degenerative lumbar spondylosis, heavy menstrual bleeding, mechanical low back pain, physiotherapy

## Abstract

A 21‐year‐old woman with dysmenorrhea, mechanical low back pain, early lumbar spondylosis, heavy menstrual bleeding, and anemia received 4 weeks of multimodal physiotherapy. Pain and function improved, but heavy bleeding created diagnostic uncertainty; menstrual flow and hemoglobin changes were interpreted cautiously and not attributed to physiotherapy alone.

## Key Clinical Message

Multimodal physiotherapy may be a useful adjunct for reducing menstrual pain and coexisting mechanical low back pain in patients with lumbar musculoskeletal dysfunction. However, heavy menstrual bleeding and anemia require careful medical evaluation, and improvements in hemoglobin or menstrual flow should not be attributed to physiotherapy alone in a single case report.

## 1 Introduction

Primary dysmenorrhea is one of the most common gynecological complaints among women of reproductive age and is characterized by cramping pain in the lower abdomen that occurs just before or during menstruation [1, 2]. It can substantially affect daily functioning, academic attendance, work productivity, and quality of life. The condition is commonly linked to increased prostaglandin production, which contributes to uterine hypercontractility, reduced uterine blood flow, and pain [3]. Conventional management usually includes non‐steroidal anti‐inflammatory drugs and hormonal therapies. However, growing evidence suggests that physiotherapy may offer a useful non‐pharmacological option, with exercise therapy, manual techniques, and electrotherapeutic modalities showing potential for pain relief in selected patients [3, 4, 5]. In addition, the frequent coexistence of musculoskeletal symptoms, particularly low back pain, indicates the importance of a broader and more integrated clinical approach [6]. In socio‐cultural settings such as Bangladesh, menstrual health concerns may remain underreported because of stigma, limited awareness, and delayed care‐seeking [7]. Published physiotherapy case literature from Bangladesh on primary dysmenorrhea with coexisting lumbar musculoskeletal dysfunction appears limited. The relationship between menstrual pain and lumbar symptoms may be explained by overlapping pelvic and lumbar pain mechanisms. Viscerosomatic convergence may allow uterine or pelvic pain to be perceived in the lumbopelvic region through shared spinal pathways. Chronic pelvic pain may also contribute to increased pelvic floor tone, lumbopelvic muscle guarding, altered posture, and movement avoidance. In patients with coexisting lumbar degenerative findings, these mechanisms may interact with mechanical pain drivers and functional limitation. Therefore, an integrated physiotherapy approach targeting pain modulation, lumbopelvic mobility, postural correction, therapeutic exercise, and patient education may be clinically reasonable. This case report describes the management of a patient with dysmenorrhea, coexisting mechanical low back pain, and MRI evidence of early degenerative lumbar spondylosis using a comprehensive physiotherapy program, with clinically meaningful short‐term improvement observed over the treatment period.

## 2 Case Presentation

A 21‐year‐old woman was referred to a tertiary rehabilitation center in Dhaka for persistent cyclic pain. Her primary symptoms included intermittent low back pain with radiation to the right gluteal region and intense cramping in the suprapubic and lower abdominal areas, consistently correlating with her menstrual cycle. The use of VAS is well‐established for quantifying menstrual pain intensity in clinical settings [8]. The symptoms had commenced six years post‐menarche and peaked during the first three to five days of menstruation, with pain severity reaching 9/10 on a VAS. Associated complaints involved profuse menstrual bleeding requiring 8–10 sanitary pads per day, pronounced fatigue, palpitations, and anxiety. Previous management relying on analgesics and sporadic medical consultations had yielded unsatisfactory results. On examination, the patient had a body mass index (BMI) of 31 kg/m^2^. Postural analysis revealed a slouched posture with a posteriorly tilted pelvis and reduced lumbar lordosis. Neurological screening was within normal limits. Lumbar range of motion was notably restricted in extension, which reproduced her familiar back pain. The Straight Leg Raise test reached 85° on the right and 90° on the left without reproduction of radicular pain below the knee. Therefore, the SLR was interpreted as negative for lumbar nerve root tension. The slightly lower right‐sided range was considered an end‐range finding, possibly related to posterior thigh/hamstring tightness rather than neural restriction [9]. Magnetic resonance imaging (MRI) of the lumbar spine indicated mild degenerative changes and disc bulges at the L3‐S1 levels. Such early degenerative changes, while uncommon, can be associated with chronic pain syndromes and postural dysfunction [10]. Blood investigations were unremarkable except for a low hemoglobin concentration of 8.5 g/dL. Psychometric assessment using the Beck Anxiety Inventory (BAI) and Fatigue Severity Scale (FSS) yielded scores of 24 and 43, respectively, indicating moderate anxiety and mild‐to‐moderate fatigue. The clinical impression was dysmenorrhea with coexisting mechanical low back pain and MRI evidence of early degenerative lumbar spondylosis. Primary dysmenorrhea was considered as a working diagnosis after gynecological assessment and normal transabdominal ultrasound; however, heavy menstrual bleeding and anemia created diagnostic uncertainty, and secondary dysmenorrhea or abnormal uterine bleeding could not be fully excluded. The patient participated in a structured, intensive physiotherapy program over four weeks, with clinical outcomes systematically evaluated at baseline and subsequent intervals.

### 2.1 Gynecological Evaluation and Exclusion of Secondary Causes

Before physiotherapy referral, the patient was evaluated by a gynecologist. A transabdominal pelvic ultrasonography was performed, which revealed a normal anteverted uterus, homogeneous myometrial echotexture with no features of adenomyosis, and normal‐sized ovaries with no evidence of endometriomas or other pelvic pathology.

The patient's history was reviewed, and she denied symptoms suggestive of endometriosis (such as dyspareunia, dyschezia, or catamenial gastrointestinal symptoms). Based on the normal imaging findings, absence of “red flag” symptoms, and a clinical history classic for primary dysmenorrhea (cyclical cramping pain starting after menarche), a working diagnosis of primary dysmenorrhea was considered; however, this diagnosis was provisional because heavy menstrual bleeding and anemia raised concern for possible secondary dysmenorrhea or abnormal uterine bleeding. Heavy menstrual bleeding requiring 8 to 10 pads/day and lasting 6 to 10 days, together with Hb 8.5 g/dL, was considered a red flag [11, 12]. Although the transabdominal ultrasound was normal, secondary causes such as endometriosis, adenomyosis, small fibroids/polyps, ovulatory dysfunction, and bleeding disorders could not be fully excluded. The patient had been prescribed mefenamic acid (500 mg as needed) by her gynecologist, which provided incomplete relief. The available clinical record did not clearly document whether oral iron supplementation, hormonal therapy, tranexamic acid, or other specific medical treatment for anemia or heavy menstrual bleeding was provided during the physiotherapy period. Nutritional counseling was provided as part of the physiotherapy program, including advice to increase iron‐rich foods, protein intake, vitamins, and hydration. Therefore, the improvement in hemoglobin during follow‐up was not interpreted as a direct effect of physiotherapy.

The chronological sequence of key clinical events, investigations, treatment milestones, and follow‐up is summarized in Table 1.

**TABLE 1:** Timeline of clinical course and management.

Time point	Clinical event
Menarche	Menstruation began at approximately 9 years of age.
Six years after menarche	Cyclical lower abdominal cramping consistent with primary dysmenorrhea began.
Subsequent course	Intermittent low back pain with radiation to the right gluteal region developed and became associated with the menstrual cycle.
Before physiotherapy referral	The patient sought intermittent medical care and used analgesics, but symptom relief remained incomplete.
Gynecology review	The patient was evaluated by a gynecologist for cyclical menstrual pain and associated symptoms.
Pelvic ultrasonography	Transabdominal pelvic ultrasonography showed a normal anteverted uterus, homogeneous myometrium, and normal‐sized ovaries, with no evidence of adenomyosis, endometrioma, or other pelvic pathology.
Diagnostic impression	Based on clinical history and normal transabdominal ultrasonography, a working diagnosis of primary dysmenorrhea was considered. However, heavy menstrual bleeding and anemia created diagnostic uncertainty, and secondary dysmenorrhea or abnormal uterine bleeding could not be fully excluded. Mefenamic acid 500 mg as needed had been prescribed, with incomplete relief.
Lumbar MRI	MRI of the lumbar spine demonstrated mild degenerative changes and disc bulges at L3 to S1.
Baseline physiotherapy assessment	The patient presented to a tertiary rehabilitation centre in Dhaka with severe menstrual pain, mechanical low back pain, postural dysfunction, restricted lumbar extension, fatigue, anxiety, and low hemoglobin. Baseline outcomes were recorded.
Week 2 review	Early clinical improvement was noted in pain intensity, sitting tolerance, and standing tolerance during the treatment period.
Week 3 review, next menstrual cycle	Menstrual pain decreased further, and the duration of severe pain shortened during the subsequent menstrual cycle.
Week 4 completion	At the completion of the 4‐week multimodal physiotherapy program, low back pain had resolved, and menstrual pain had reduced substantially, with improvements in functional tolerance and secondary outcomes.
Follow‐up cycle	Improvement was sustained during the follow‐up menstrual cycle, with no recurrence of low back pain and only mild residual menstrual pain.

## 3 Management and Outcome

A bespoke, multimodal physiotherapy regimen was implemented, as detailed in Table 2. The intervention was strategically divided to address both musculoskeletal and gynecological components simultaneously. In this multimodal plan, core therapies (therapeutic exercises and education) were prioritized to correct underlying postural and mobility issues, while adjunctive modalities (heat, TENS, taping) were used to relieve pain and enhance comfort.

**TABLE 2:** Multimodal physiotherapy intervention protocol.

Week/Phase	Core interventions	Adjunct interventions	Dosage/frequency
Week 2 (clinic‐based phase; 6 sessions)	Repetitive lumbar extension in prone lying; hip and trunk stretching; pelvic floor muscle exercises in supine lying; postural education; aerobic training	Soft tissue mobilization to the lower abdomen and lumbosacral region; superficial heat therapy; TENS	Lumbar extension: 10 repetitions every 2 h as tolerated; stretching: 3–5 repetitions, 3 times/day; pelvic floor exercises: 10 repetitions with 10‐s hold, 2 times/day; soft tissue mobilization: 5–10 min/session; heat therapy: 20–30 min/session, up to 3 times/day; TENS: 50 Hz, 20 min/session; aerobic exercise: 15–20 min walking plus 10 min static cycling
Week 3 (progression phase; 3 clinic sessions)	Repetitive lumbar extension; progressed hip, trunk, and pelvic girdle stretching with therapist overpressure as tolerated; pelvic floor training progressed to sitting and standing; aerobic progression; postural correction	Soft tissue mobilization; superficial heat therapy; TENS	Lumbar extension: 10 repetitions every 2 h as tolerated; stretching: 5–8 repetitions, 3 times/day; pelvic floor exercises: 15–20 repetitions with 10‐s hold, 3 times/day; heat therapy: 20–30 min/session, 3–5 times/day as needed; TENS: 50 Hz, 20 min/session; aerobic exercise: 30–45 min walking, 5 days/week
Week 4 (functional integration phase; 6 clinic sessions)	Repetitive lumbar extension; functional stretching; pelvic floor training integrated into daily activities; aerobic conditioning; adherence monitoring and home exercise review	Soft tissue mobilization; superficial heat therapy; TENS as needed	Lumbar extension, stretching, pelvic floor exercise, and postural correction were continued with progression as tolerated; heat therapy: 20–30 min/session as needed; TENS: 50 Hz, 20 min/session as needed; aerobic exercise: 15–20 min walking plus 15 min static cycling
Menstrual week after completion of Week 4	Exercise intensity was temporarily reduced during the first 3 days of menstruation according to patient preference and symptom tolerance	Soft tissue mobilization; superficial heat therapy; TENS; Kinesio taping to the lower abdomen and lumbar region	Soft tissue mobilization and heat therapy were used for symptomatic relief; TENS: 50 Hz, 20 min/session as needed; Kinesio taping was applied intermittently for 3–5 days during the symptomatic phase

*Note:* Patient education throughout the program: The patient received education on posture, activity modification, lifting precautions, menstrual pain self‐management, stress management, hydration, vaginal hygiene, and home exercise adherence. Nutritional counseling was provided, including advice to increase iron‐rich foods, protein intake, vitamins, and hydration, and to reduce excessive sugar and caffeine intake. TENS was applied at 50 Hz for 20 min/session, as documented in the physiotherapy treatment protocol.

**FIGURE 1:**
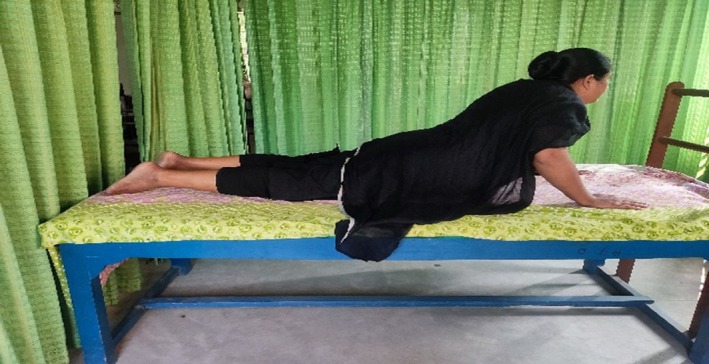
Repetitive lumbar extension exercise performed in prone lying as part of the physiotherapy program to address mechanical low back pain and restricted lumbar extension.

For the lumbar dysfunction, the management capitalized on an identified directional preference using the McKenzie method [13]. The patient was instructed in repetitive lumbar extension exercises in prone lying (Figure 1) to be performed throughout the day. Postural re‐education was emphasized, with specific advice to avoid sustained flexion.

**FIGURE 2:**
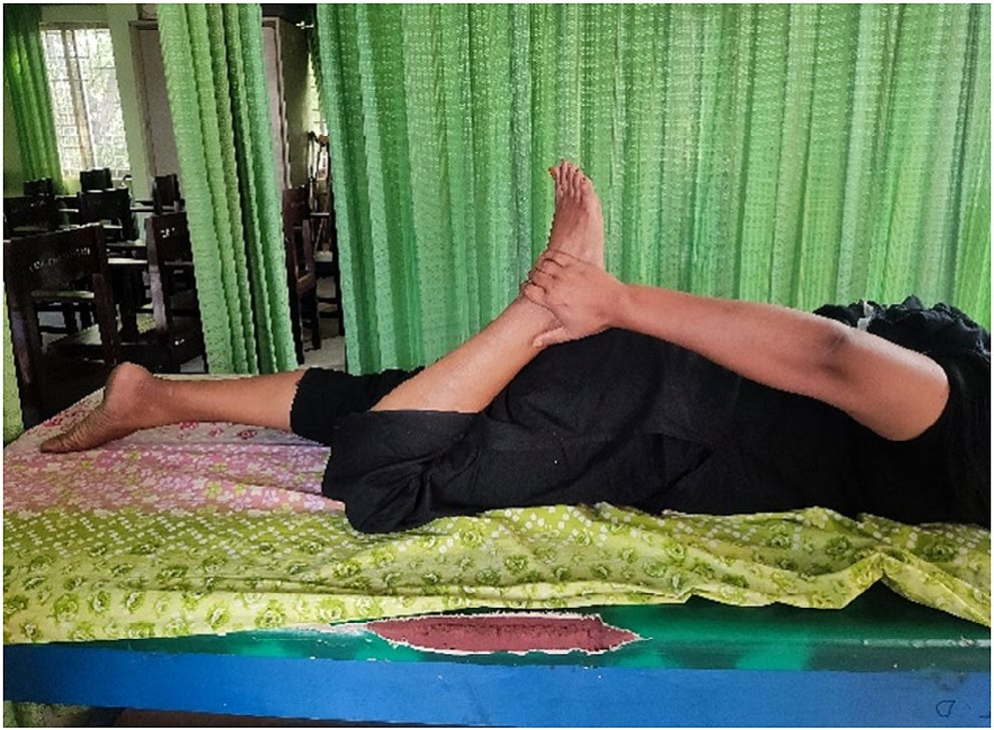
Hip stretching exercise prescribed to reduce lumbopelvic muscle tightness and improve flexibility during the rehabilitation program.

**FIGURE 3:**
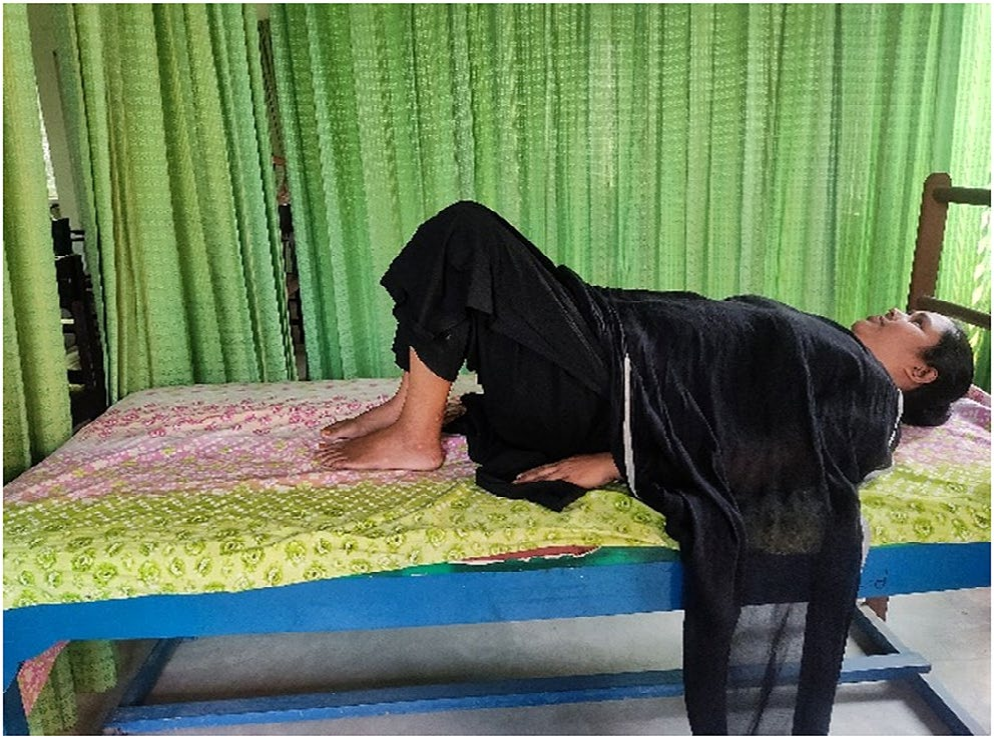
Pelvic floor muscle contraction exercise initiated in the supine position as part of the strengthening component of the intervention.

**FIGURE 4:**
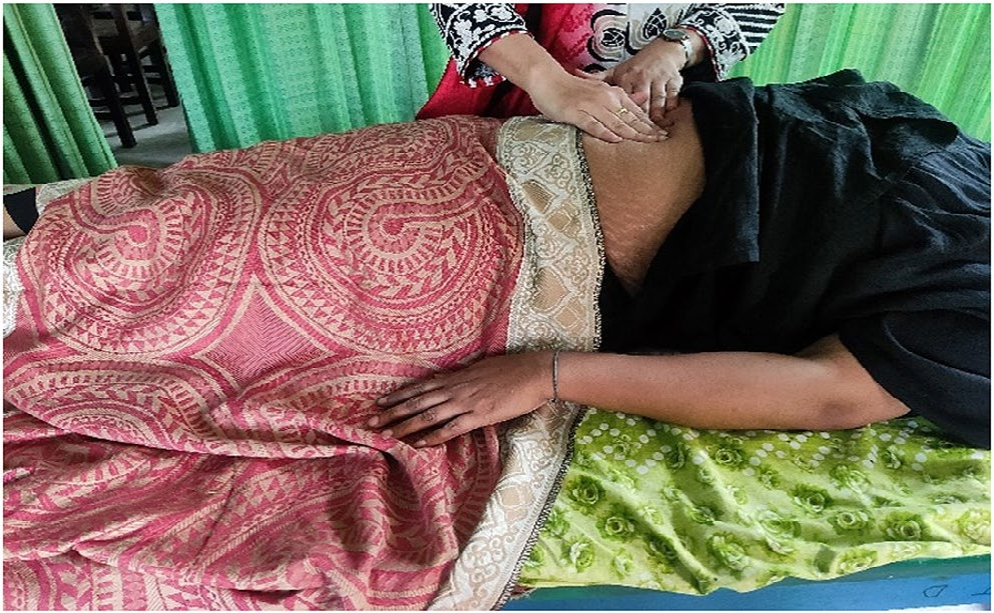
Soft tissue massage applied to the lower abdominal region as an adjunctive intervention during symptomatic periods.

**FIGURE 5:**
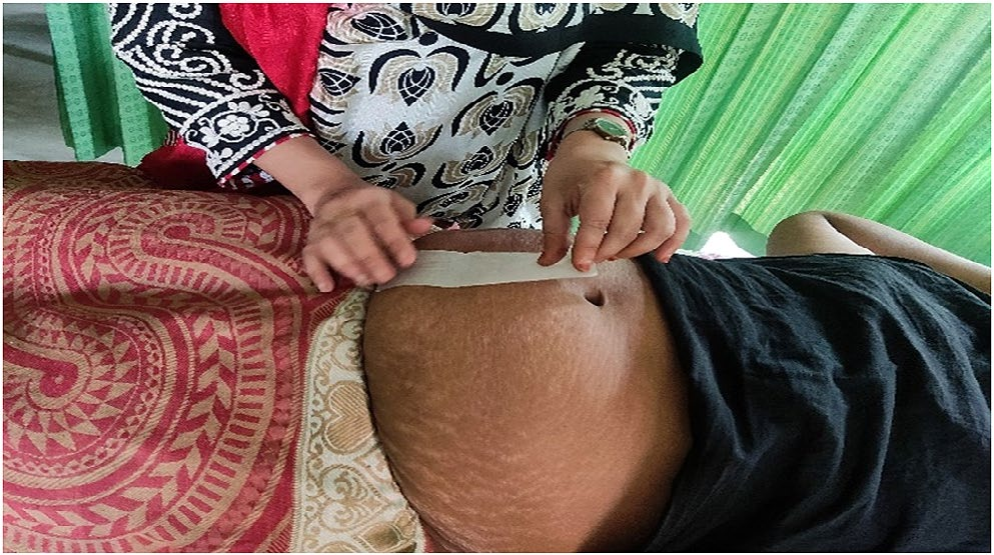
Kinesio taping applied to the lower abdominal region as an adjunctive pain‐modulating intervention during menstruation.

**FIGURE 6:**
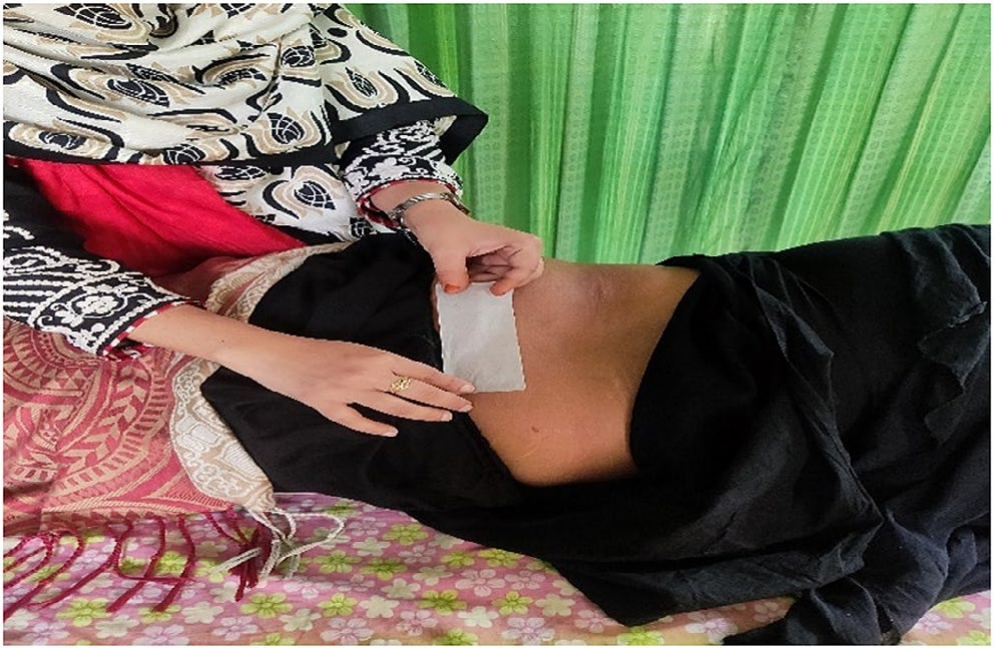
Kinesio taping applied to the lower back to support pain relief and tissue function during the symptomatic phase.

**FIGURE 7:**
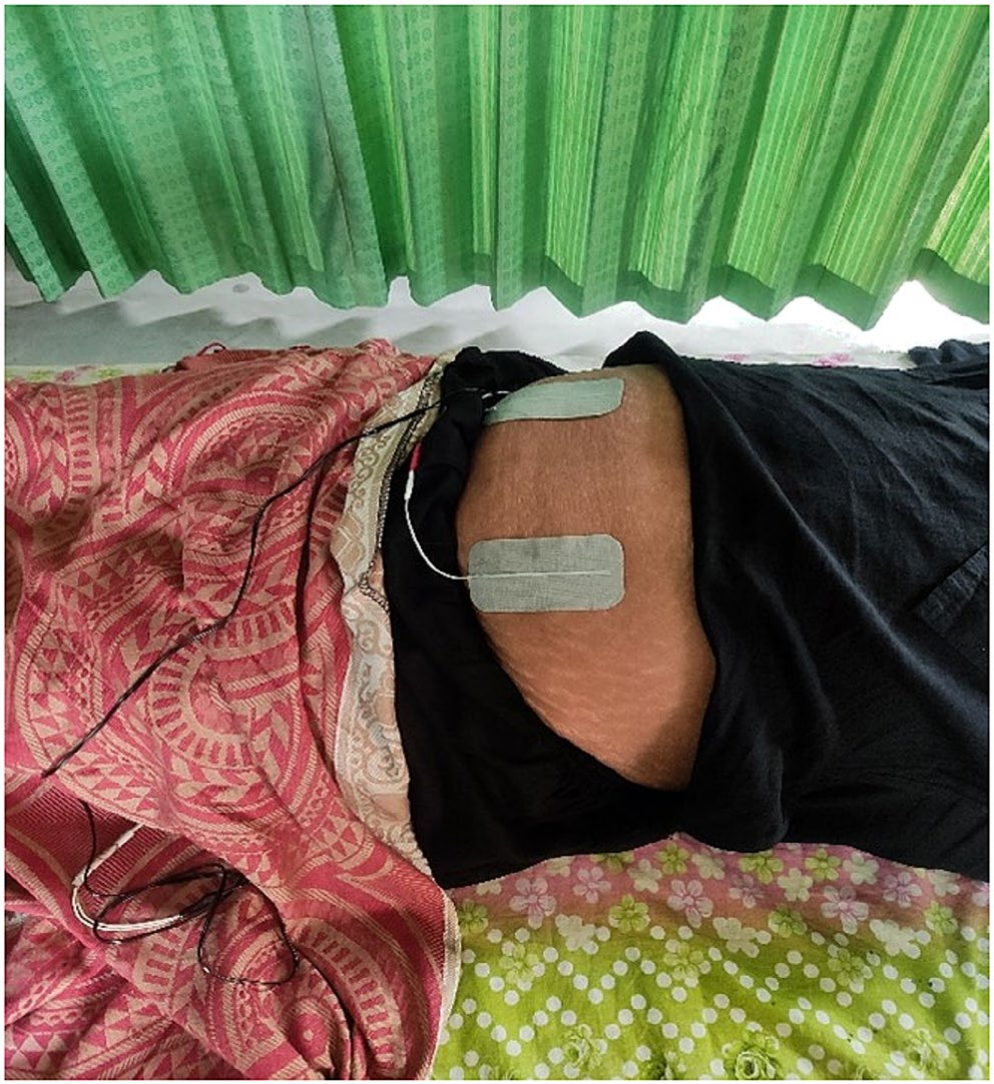
Transcutaneous electrical nerve stimulation (TENS) applied using a four‐electrode setup to the lower back and lower abdominal region.

To address the dysmenorrhea, a regimen of stretching exercises for the hip (Figure 2), abdominal, and pelvic girdle muscles was prescribed. Pelvic floor muscle strengthening (Kegel exercises) was initiated in a supine position (Figure 3) and progressively integrated into functional positions, as strengthening this musculature may improve pelvic circulation and support, contributing to pain reduction [14]. Adjuvant therapies included soft tissue massage to the lower abdomen (Figure 4) and the lumbosacral region. The application of Transcutaneous Electrical Nerve Stimulation (TENS) using a four‐electrode setup on the back and lower abdomen was also employed, alongside consistent use of superficial heat therapy. Kinesio taping was applied to the lower abdomen (Figure 5) and lower back (Figure 6) during the symptomatic phase to facilitate pain relief and tissue function through neurosensory stimulation and improved microcirculation. As a non‐invasive, culturally acceptable modality, it could enhance patient compliance (Figure 7).

**FIGURE 8:**
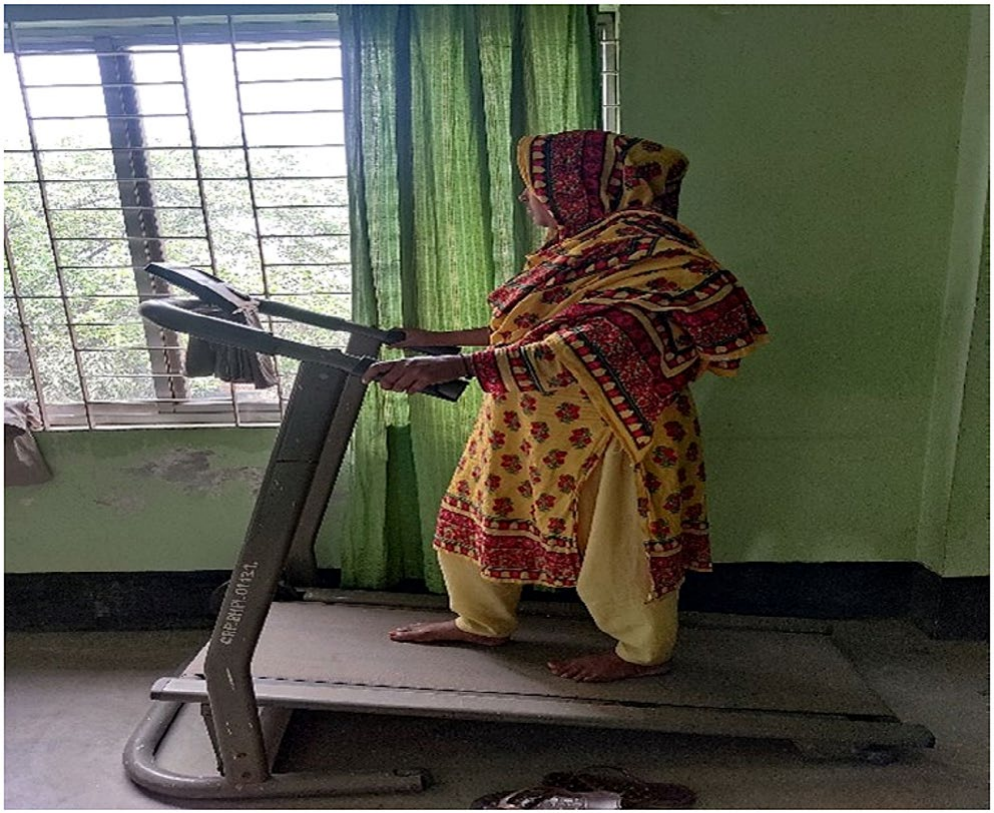
Aerobic exercise.

To support her overall health, a moderate‐intensity aerobic exercise program was introduced, including the use of static cycling (Figure 8). Crucially, the patient received comprehensive counseling on nutritional modifications to improve iron and protein intake, stress management techniques, and personal hygiene practices.

The patient demonstrated clinically meaningful short‐term improvement across pain and functional outcomes, as summarized in Table 3. Improvements in menstrual flow, fatigue, anxiety, palpitations, and hemoglobin were recorded as secondary observations. However, these secondary changes were considered multifactorial and were not attributed solely to the physiotherapy intervention.

**TABLE 3:** Clinical outcomes across baseline, treatment, and follow‐up.

Primary outcomes					
Outcome measure	Baseline	Week 2	Week 3 (next menstrual cycle)	Week 4	Follow‐up cycle
Low back pain (VAS)	5 cm usual pain; 9 cm during menstruation	6 cm	2–3 cm	No pain	No pain
Lower abdominal/groin pain (VAS)	9 cm	7 cm	3–5 cm	3 cm	3 cm, mild
Duration of severe pain	3–5 days	3–5 days	2 days, mild	2 days, mild	2 days, mild
Sitting tolerance	< 10 min	> 15 min	> 40 min	60 min	> 60 min
Supine lying tolerance	< 5 min	No change	> 10 min	10–15 min	Functional
Standing/bending tolerance	< 20 min with severe pain	> 30 min	> 30 min	> 1 h	Functional with minimal discomfort

Secondary outcomes					
Outcome measure	Baseline	Week 2	Week 3	Week 4	Follow‐up cycle
Sanitary pads/day, self‐reported	8–10	7–8	5–6	5–6	5–6
Heavy flow duration	6–10 days	3–4 days	3–4 days	2–3 days	2–3 days
Fatigue Severity Scale (FSS)	43	—	—	22	22
Beck Anxiety Inventory (BAI)	24	—	—	18	18
Pain medication use	Mefenamic acid 500 mg as needed; antispasmodic use reported but name not documented	Same as baseline	NSAID only	Paracetamol as needed	Paracetamol as needed
Hemoglobin	8.5 g/dL	—	—	10 g/dL	10 g/dL
Palpitations	Present	Present	Reduced	Absent	Absent
Treatment adherence	Not applicable	3 clinic sessions/week	4 sessions/week, including clinic and home program adherence	4 sessions/week, including clinic and home program adherence	Home program continued, self‐reported

Abbreviations: BAI = Beck Anxiety Inventory; FSS = Fatigue Severity Scale; NSAID = non‐steroidal anti‐inflammatory drug; VAS = Visual Analogue Scale. “—” indicates that data were not collected at that time point. The follow‐up cycle refers to the next menstrual cycle after completion of the 4‐week physiotherapy program. No adverse or unanticipated responses were reported during the treatment period. Sanitary pad use was self‐reported and was not measured using a validated pictorial blood loss assessment chart. Hemoglobin change was recorded as a secondary clinical observation and was not considered a direct physiotherapy outcome. The available clinical record did not clearly document whether oral iron supplementation or other specific anemia treatment was provided during the physiotherapy period.

## 4 Discussion

This case describes short‐term clinical improvement following a holistic physiotherapy protocol for a complex presentation of PD with comorbid LBP. The observed positive outcomes resonate with existing scientific literature. A robust Cochrane systematic review has affirmed the role of regular exercise in alleviating the intensity of menstrual pain [4]. The prescribed stretching and pelvic floor exercises are theorized to mitigate referred pain patterns and enhance pelvic circulation, thereby facilitating the clearance of algogenic substances like prostaglandins [3, 14]. The incorporation of electrophysical agents provided effective, non‐pharmacological analgesia. TENS is known to modulate pain perception through the gate control theory and endogenous opioid release [15], while thermotherapy reduces muscle tension and promotes relaxation [16]. The use of Kinesio taping, aimed at facilitating cutaneous neural feedback and improving microcirculation, may have contributed to pain reduction and tissue recovery [17]. Systematic reviews and randomized trials have shown that aerobic conditioning and therapeutic exercise are the intervention components with the best evidence for reducing dysmenorrhea discomfort [4]. There is also some evidence that electrophysical treatments like thermotherapy and TENS can provide short‐term pain relief [16, 17]. Kinesio taping, on the other hand, was regarded as an adjunct rather than a major intervention because the research supporting it is still scant and somewhat debatable.

A pivotal element of this management was its integrated, biopsychosocial approach, which concurrently addressed the gynecological pain, musculoskeletal dysfunction, and associated psychological distress. This is particularly salient in the Bangladeshi context, where deep‐seated cultural taboos often prevent open discussion of menstrual health [7]. This case demonstrates that with sensitive and comprehensive care, these barriers can be overcome.

The significant improvement in secondary outcomes like anxiety and fatigue underscores the broader biopsychosocial impact of managing chronic pain conditions [18]. However, improvements in menstrual bleeding patterns and hemoglobin levels cannot be conclusively attributed to physiotherapy alone and may reflect broader lifestyle modifications and natural variability. Furthermore, this case provides a practical, multimodal protocol that can be replicated in low‐resource settings and demonstrates a model for overcoming cultural barriers to care for an often‐stigmatized condition. Several limitations must be acknowledged. First, this was a single case report using a multimodal intervention; therefore, the relative contribution of exercise, education, manual therapy, heat, TENS, taping, aerobic conditioning, and nutritional advice could not be separated. Second, the short follow‐up period limits conclusions about the durability of treatment effects. Third, there was diagnostic uncertainty regarding the classification of dysmenorrhea. Although the patient's cyclical pain pattern and normal transabdominal ultrasonography supported a working diagnosis of primary dysmenorrhea, the presence of heavy menstrual bleeding, prolonged menstrual flow, and a hemoglobin concentration of 8.5 g/dL raised concern for possible secondary dysmenorrhea or abnormal uterine bleeding [11, 12]. Further investigations, such as transvaginal ultrasonography, laboratory evaluation for anemia, coagulation screening, or laparoscopic assessment, were not performed or were not available in the clinical record. Therefore, secondary gynecological or systemic causes could not be fully excluded. Fourth, the available record did not clearly document whether oral iron supplementation or other specific medical treatment for anemia was provided during the physiotherapy period. For this reason, the observed improvement in hemoglobin and menstrual flow should be interpreted cautiously and should not be attributed to physiotherapy alone. Further controlled studies with clearer diagnostic workup, longer follow‐up, and separate evaluation of individual treatment components are needed.

### 4.1 Patient Perspective

The patient reported that the physiotherapy program helped reduce both her menstrual pain and low back pain and made her daily activities easier to perform. She felt more comfortable sitting, standing, and moving during her menstrual cycle compared with before treatment. She also stated that the education, exercises, and supportive care increased her confidence in managing her symptoms without depending only on medication. Overall, she was satisfied with the treatment and felt encouraged by the improvement achieved over the course of therapy.

## 5 Conclusion

A structured multimodal physiotherapy program may be a useful adjunct for managing dysmenorrhea with coexisting mechanical low back pain and lumbar musculoskeletal dysfunction. In this case, improvements were observed in pain and functional tolerance over a short treatment period. However, because heavy menstrual bleeding and anemia were present, secondary dysmenorrhea or abnormal uterine bleeding could not be fully excluded. Therefore, changes in menstrual flow and hemoglobin should be interpreted cautiously. Further studies with clearer diagnostic workup, controlled designs, and longer follow‐up are needed.

**Learning Points**.

- Dysmenorrhea may coexist with mechanical low back pain and lumbar degenerative findings, requiring both gynecological and musculoskeletal assessment.
- Heavy menstrual bleeding and anemia should be treated as important red flags when classifying dysmenorrhea as primary.
- Multimodal physiotherapy may help reduce pain and improve function, but changes in hemoglobin or menstrual flow should not be attributed to physiotherapy alone.
- Case reports should clearly state diagnostic uncertainty, confounding factors, and limitations

## Author Contributions

**Md Rifat Haidar:** writing – review and editing, writing – original draft. **Nadia Afrin Urme:** conceptualization, project administration, writing – review and editing. **Ranu Islam:** investigation, validation, resources, writing – review and editing. **Fabiha Alam:** investigation, writing – review and editing, resources. **Asma Islam:** methodology, writing – review and editing, supervision. **Polok Halder:** methodology, supervision, writing – review and editing, writing – original draft, project administration. **Md Waliul Islam:** writing – review and editing, funding acquisition, formal analysis, validation.

## Funding

The authors have nothing to report.

## Ethics Statement

Ethical approval for this case report was obtained from the Departmental Review Committee of the Bangladesh Health Professions Institute, Centre for the Rehabilitation of the Paralyzed, Bangladesh. Written informed consent to participate was obtained from the patient before data collection and treatment procedures.

## Consent

Written informed consent was obtained from the patient for publication of this case report and any accompanying images or clinical details. The patient was informed that all identifying information would be removed to preserve anonymity.

## Conflicts of Interest

The authors declare no conflicts of interest.

## Data Availability

The data that support the findings of this study are available from the corresponding author upon reasonable request.
